# Comparative Assessment of Groundwater Quality in the Tangshan Region of the People’s Republic of China and Similar Areas in the U.S.

**DOI:** 10.1100/tsw.2001.293

**Published:** 2001-11-10

**Authors:** Joseph L. Domagalski, Lin Chao, Zhou Xinquan

**Affiliations:** U.S. Geological Survey, Sacramento, CA 95819, USA

**Keywords:** water quality, agriculture, nitrate, aquifers, United States, People’s Republic of China, Tangshan, isotopes, tritium

## Abstract

Groundwater quality with respect to nitrate, major inorganic constituents, stable isotopes, and tritium was assessed in the agricultural Tangshan region in the Hai He River Basin of the People’s Republic of China and compared with three regions in the U.S.: the Delmarva Peninsula of Delaware, Maryland, and Virginia; the San Joaquin Valley of California; and the Sacramento Valley of California. The China and U.S. regions are similar in size and land use, but have different climatic conditions and patterns of water use for irrigation. The Tangshan region has been in agricultural production for a much longer time, probably several centuries, than the three U.S. regions; however, the widespread use of synthetic fertilizers and other soil amendments probably started at a similar time in all four regions. In all four regions, median nitrate concentrations were generally below the U.S. drinking water standard of 10 mg/l of nitrate as nitrogen. However, higher concentrations and a greater range were evident for the Tangshan region. In the water samples collected from a shallow aquifer in the Tangshan region (over 25% of all samples), nitrate concentrations exceeded the Chinese standard of 20 mg/l, whereas few comparative samples (2.6%) collected in the U.S. exceeded 20 mg/l. In Tangshan, relatively low nitrate, which is indicative of uncontaminated background concentrations, was measured in older water of deeper wells. Recently recharged water was detected in wells drilled as deep as 150 m. Nitrate concentrations above background levels were also measured in water samples from these wells. In addition to nitrate, the agricultural area of the Tangshan region has been affected by elevated total dissolved solids and iron, the latter attributed to widespread application of animal wastes and sewage deposited on the land surface, which lead to oxygen depletion in the subsurface environment and dissolution of iron. The elevated total dissolved solids of the Tangshan study area could not be attributed to any one process.

## INTRODUCTION

The use of nitrogen fertilizer, either in chemical form or as manure, has resulted in the degradation of water supplies used for drinking water, principally because of the accumulation of

This paper is an abbreviated version of the U.S. Geological Survey Professional Paper 1647, "Comparative Water Quality Assessment of the Hai He River Basin in the People's Republic of China and Similar Areas in the U.S.," written by Domagalski et al. and published in 2001.

residual nitrate. Many countries regulate nitrate levels in drinking water to prevent human health problems. Elevated levels of nitrate are known to cause blue-baby syndrome in young children and may increase the risk of cancer through lifelong exposure. Groundwater in unconfined aquifers is particularly susceptible to contamination because of the application of fertilizers to soil. Research has determined the susceptibility of various aquifers to contamination, and management techniques to reduce or eliminate contaminants have been developed[1,2].

To help address problems caused by fertilizer contamination in one agricultural region of the People's Republic of China, a cooperative study with the U.S. Geological Survey was undertaken to understand the distribution of nitrate in groundwater and to compare the water quality with similar areas of the U.S. The area chosen in the People's Republic of China is the Tangshan study area, which is located about 160 km southeast of Beijing and encompasses about 13,470 km$^2$ (Fig. 1). Three regions were chosen in the U.S. for comparison: the Sacramento Valley (16,392 km$^2$) in the Sacramento River Basin, California; the San Joaquin Valley (31,216 km$^2$) in the San Joaquin-Tulare Basins, California; and the Delmarva Peninsula of Delaware, Maryland, and Virginia (15,540 km$^2$) (Fig. 2).

The Tangshan study area is in the Hai He River Basin (Fig. 1) and encompasses about 110 million square hectometers of farmland (Lin Chao, Hai He River Water Conservancy Commission, unpublished data, 1998). More than 118 million people reside in the basin. Demand for available water supplies is great despite recent construction of reservoirs built to improve supply and quality. Groundwater is used to irrigate agricultural land, but current rates of extraction put a stress on this resource[3]. Crops grown in the Tangshan study area include wheat, corn, rice, sorghum, cotton, and peanuts.

Crops also are extensively irrigated in both the Sacramento and San Joaquin Valleys[4,5]. Crops in these areas are diverse and include rice, orchards, and row crops. In contrast, the Delmarva Peninsula[6] has rainfall during the growing season, and irrigation is only used during periods of drought. Crops in the Delmarva area include corn, soybeans, row crops, and animals (confined feeding operations).

## EXPERIMENTAL METHODS AND PROCEDURES

In 1996, 111 wells were selected and sampled in the Tangshan study area (Fig. 3). The strategy was to select a shallow and a deep well at each location. The shallow wells tapped an upper aquifer that has a thickness of 20 to 140 m, and the deeper wells tapped the aquifer or aquifers below. The well-selection strategy was based on the concept of a regional groundwater survey, as described elsewhere[7]. More specific information on the well-selection strategy for the study areas of the U.S. also is available and described in other studies[4,5,6,8]. Water samples were analyzed for major elements (Na, K, Ca, Mg, Cl, and $SO_4$), nutrients ($NO_3$, $NO_2$, $NH_4$, and $PO_4$), specific conductance, pH, alkalinity, dissolved oxygen, and select trace elements. In the Tangshan

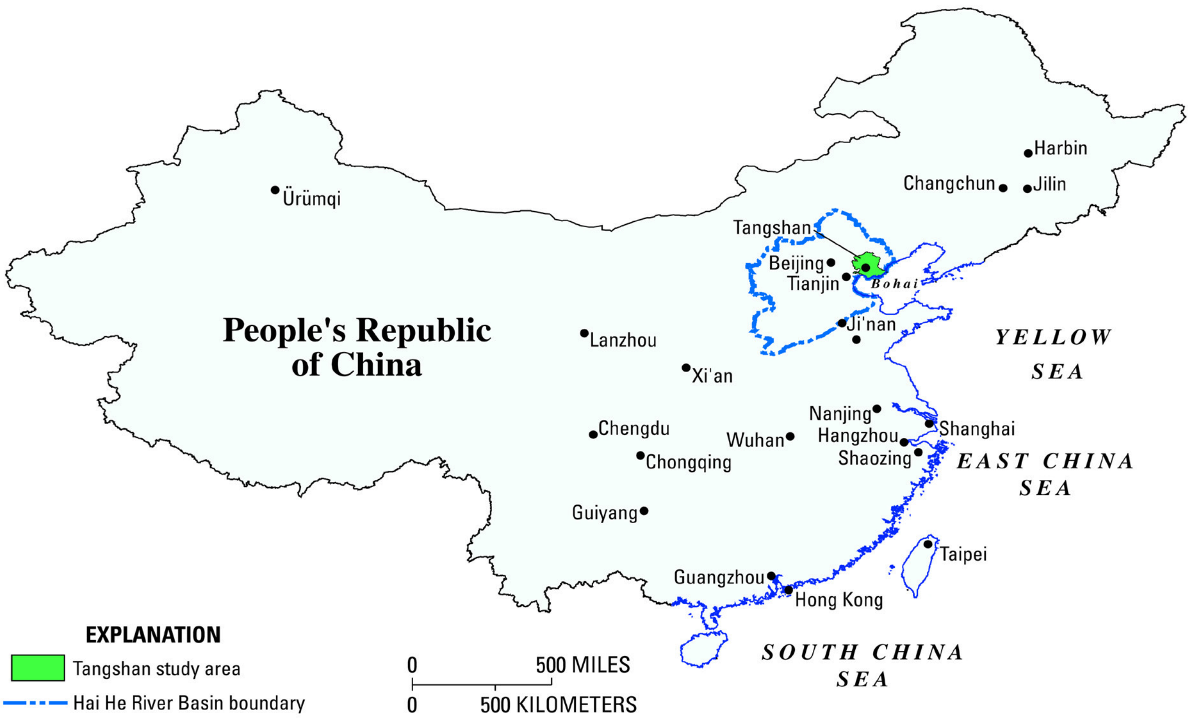

**FIGURE 1.** Map of People's Republic of China showing the Hai He River Basin and the Tangshan study area.

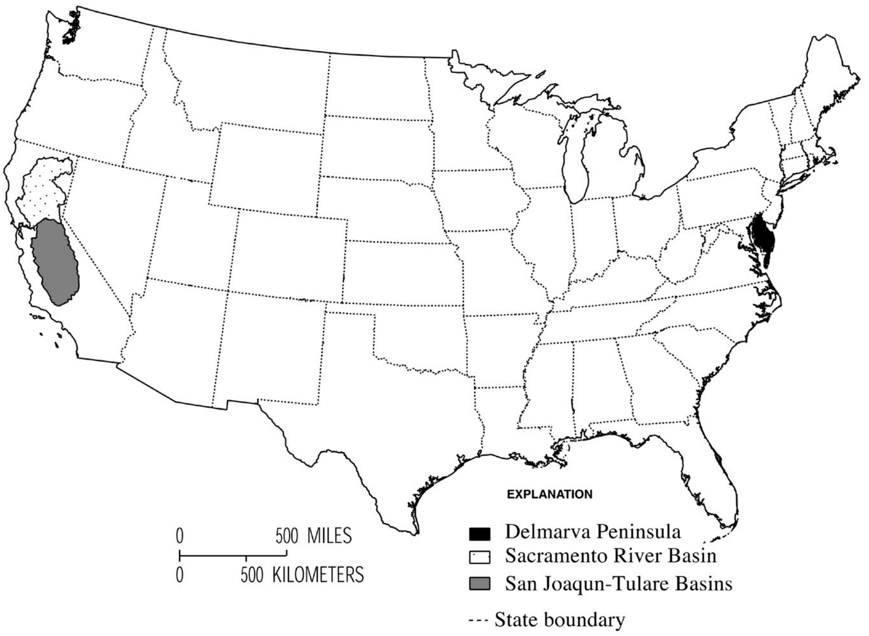

**FIGURE 2.** Map of U.S. showing the Delmarva Peninsula, the Sacramento River Basin, and the San Joaquin–Tulare Basins.

aquifer, stable isotope ratios of deuterium ($^2$H) to hydrogen ($^1$H), oxygen-18 ($^{18}$O) to oxygen-16 ($^{16}$O) and, in nitrate molecules, nitrogen-15 ($^{15}$N) to nitrogen-14 ($^{14}$N) and $^{18}$O to $^{16}$O were measured in selected water samples to better understand the groundwater flow system and sources of nitrate. Analyses of oxygen and hydrogen isotopes[9] and nitrogen isotopes[10] followed established methods and results are expressed as per mill (parts per thousand, using the symbol δ).

In the Tangshan study area (Fig. 3), stable isotope samples were collected from groundwater along four selected transects chosen to provide information on the regional distribution of stable isotope ratios of water in this aquifer system. Where possible, a shallow and a deep well were sampled at each of the transect sites. Transect 1, closest to the Luan He River, was chosen to determine the relation between the groundwater system and the largest river. Transect 2, located near the Qinglong He River, was chosen to compare stable isotopes of an ephemeral stream with those of nearby groundwater. Transects 3 and 4 were chosen to obtain greater aerial coverage over the study area.

Recharge dates of groundwater were determined using three methods: tritium ($^3$H), ratio of $^3$H to the daughter product of tritium decay [helium-3 ($^3$He)], and chlorofluorocarbon compounds (CFC). The presence of $^3$H indicates groundwater recharged since the early 1950s, whereas the $^3$H/$^3$He and CFC methods provide better temporal resolution for groundwater recharged after the early 1950s[11,12].

## RESULTS

The stable isotope pattern of groundwater in the Tangshan study area (Fig. 4) indicates that groundwater of the mountain and plain regions is very similar to that of the Luan He River. In the coastal region, groundwater is isotopically more variable because seawater has infiltrated the groundwater system. The δ $^{18}$O values in the wells sampled in the mountain and plain regions, and in the Luan He River, tend to be close to –8 to –8.5 per mill, and the δ$^2$H value is close to –60 per mill. One sampling site near the Luan He River (location Lao6, Fig. 3) was different: the shallow well was slightly enriched in the heavier isotopes (δ$^2$H, –58; δ$^{18}$O, –7.86), but the deeper well (δ$^2$H, –75; δ$^{18}$O, –9.61) was more depleted in the heavier isotopes. This was the closest sampling point to the Luan He River. The difference in isotope values may reflect changes in the isotopic chemistry of the Luan He River, especially prior to the construction of reservoirs. The stable isotopes of the Qinglong He River were relatively enriched in the heavier isotopes, which would be expected for an ephemeral stream, such as the Qinglong He River, owing to evaporation. In wells sampled closest to the Qinglong He River, water from the upper aquifer is slightly enriched in the heavier isotopes, suggesting that some recharge from partially evaporated stream water has occurred.

The general uniformity of isotope ratios observed in most of the groundwater sampled from the mountain and plain regions and the Luan He River suggests a relatively uniform source of recharge water, probably the result of the precipitation pattern. Rainfall moisture originates over the Pacific Ocean and rain having a similar pattern of stable isotopes falls over the plain and mountain regions.

Boxplots of specific conductance for the wells of each study area are shown in Fig. 5. Groundwater sampled from the Delmarva Peninsula exhibits the lowest median specific conductance (203 µs/cm), probably due to the interaction of dilute meteoric water with the relatively insoluble quartz sand of the aquifer. In contrast, groundwater sampled from the San Joaquin Valley has the highest specific conductance, having a median value of 704 µs/cm. The higher specific conductance of groundwater of the San

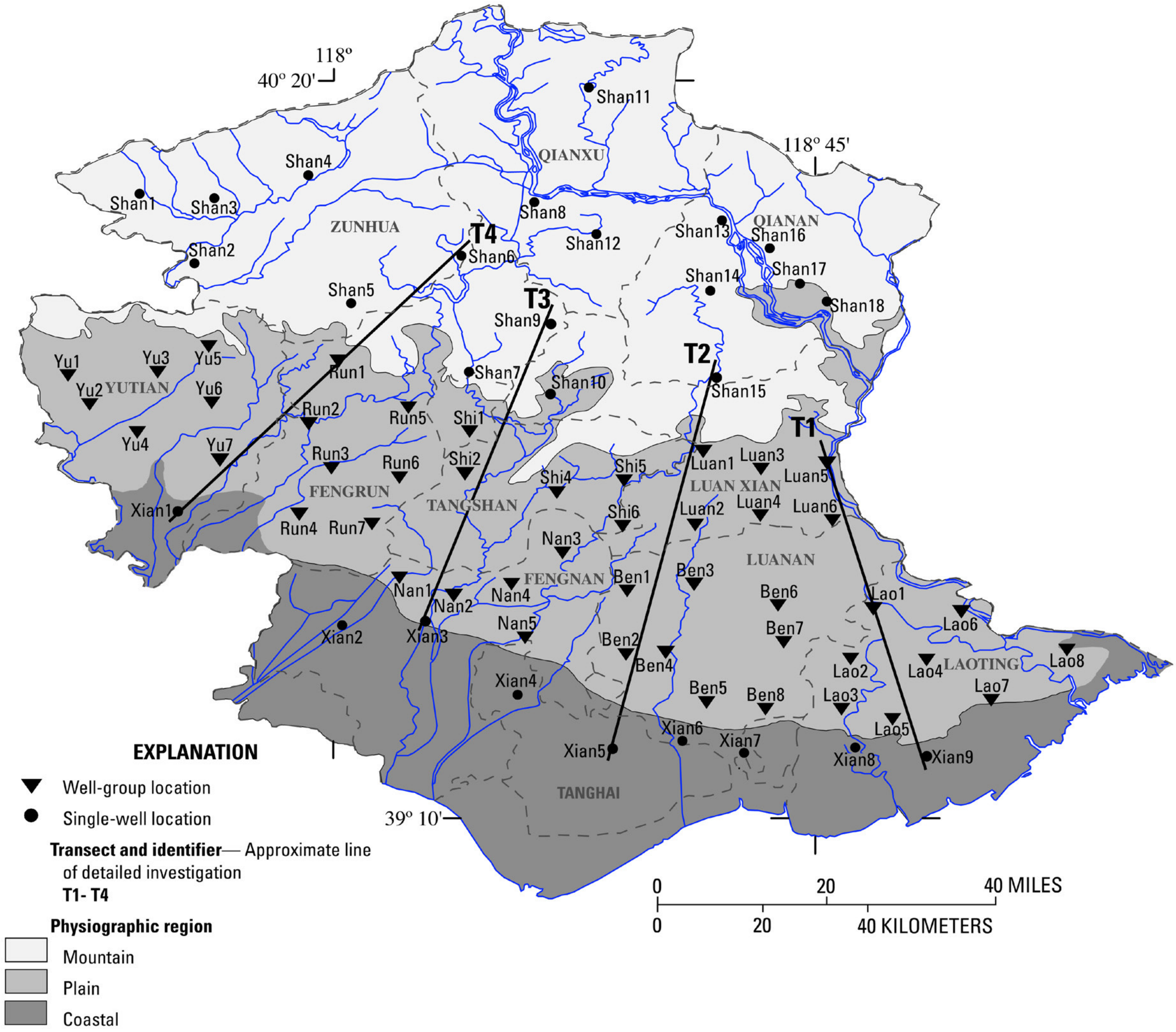

**FIGURE 3.** Tangshan study area showing locations of wells, transects (T1 through T4) for detailed investigations, county boundaries, and physiographic regions. A well group consists of two or more wells.

Joaquin Valley can be attributed to relatively high levels of dissolved solids in some of the recharge water and irrigation in a semiarid environment, the latter of which leaves behind salts as water evaporates. The specific conductance of sampled groundwater of the Sacramento Valley is most similar to that of the Tangshan study area, with a median specific conductance of 612 and 625 µs/cm, respectively. Boxplots of specific conductance for wells of the physiographic regions of the Tangshan study area (mountain, plain, and coastal), including the upper and lower aquifer of the plain region, are shown in Fig. 6. The lowest median specific conductance is from wells in the mountain region, where most groundwater recharge is under natural conditions and near the start of flow path. The highest median specific conductance is from wells in the upper aquifer of the plain region. In the lower aquifer, specific conductance is significantly lower, which suggests that salts in the upper aquifer are a result of land use. In the coastal region, the specific conductance of the groundwater is similar to that of the lower aquifer of the plain region, except where affected by seawater intrusion.

Concentrations of iron in groundwater provide information on the state of oxidation or reduction of the groundwater and indicate where reduction of nitrate to nitrogen gas may occur[13]. Iron is insoluble when oxidized, but is much more soluble in the reduced state. The greatest range in iron concentrations (1 to

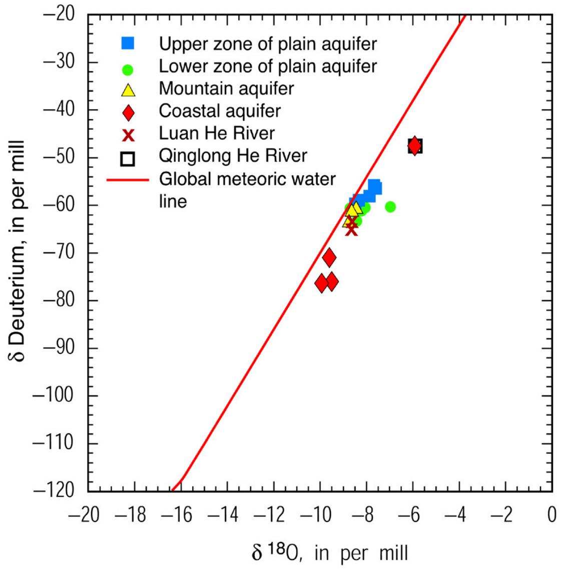

**FIGURE 4.** Boxplots of stable isotopes δ deuterium ($^{2}H$) vs. $^{18}O$ of groundwater in the Tangshan study areas and the Qinglong and Luan He Rivers.

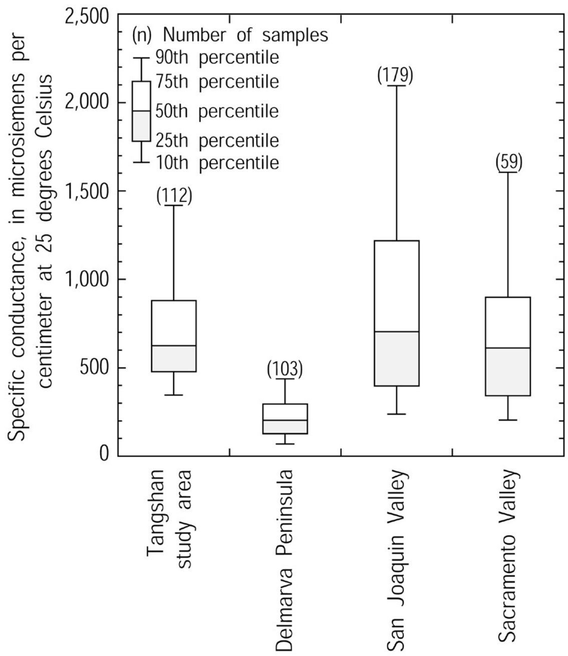

**FIGURE 5.** Boxplots of specific conductance for wells within each study area.

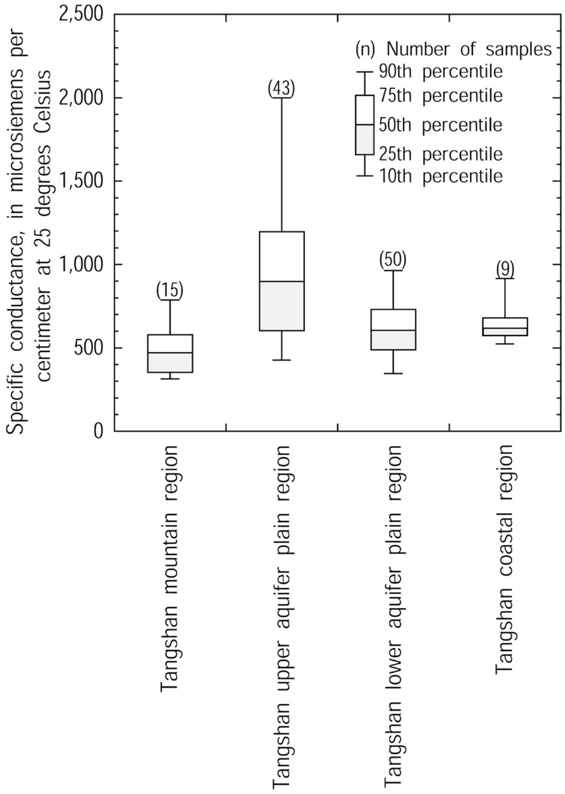

**FIGURE 6.** Boxplots of specific conductance for wells in various physiographic regions of the Tangshan study area.

6,920 µg/l), for the Tangshan study area was in the upper aquifer of the plain region, which has a median concentration of 290 µg/l. The median iron concentration for the lower aquifer of the plain region is 100 µg/l. Median iron concentrations of the coastal region of the Tangshan study area are similar to that of the upper aquifer of the plain region. However, the higher iron concentrations in the upper aquifer of the plain region was unexpected and suggest that some processes are affecting the redox potential of the groundwater, allowing the iron from the aquifer material to go into solution. Normally, higher concentrations of iron in a lower aquifer are expected because the deeper groundwater is older and has had more time for oxygen depleting reactions to occur. The wide range and higher concentrations of iron in the upper aquifer suggest that oxygen is being removed from the groundwater by some process. That process may be related to the use of manure on agricultural land or to the input of sewage waste over the land surface, which may cause a decrease in the oxidation/reduction potential of the groundwater, allowing more iron to dissolve.

Controls on iron concentrations in the study areas of the U.S. appear to be more related to geological factors such as soil texture[6,8]. Groundwater overlain by coarse-grained sediment tends to have higher oxygen levels and hence low or nondetectable iron, whereas groundwater overlain by fine-grained soils tends to have lower to nondetectable dissolved oxygen. In addition, anoxic groundwater can result in the reduction of nitrate to nitrogen gas, as long as the required microbial population and sufficient carbon are present. Principal component analyses of the groundwater chemistry for all study areas show an inverse relation between nitrate and iron concentrations for these groundwaters.

Prior to this study, there had been no measurements of $^{3}H$ or any attempts to establish the age of groundwater in the Tangshan aquifer study area. The wells located along transects 1 and 2 (Fig. 3) were selected for these analyses. The detection of $^{3}H$ or CFC to a well depth of 150 m suggests that recently recharged water occurs to that depth in the lower aquifer of the plain region. In the coastal region, groundwater sampled from depths of 212 and 230 m did not have measurable $^3$H. Recent water in relatively deep parts of the aquifer (150 m below land surface) suggests that the groundwater system is rapidly recharged by some combination of meteoric water (during the Asian monsoon) and infiltration from rivers and irrigation. Groundwater pumpage from deeper wells helps to draw the recently recharged water to deeper levels. Soil contaminants, or contaminants at shallow depths, such as nitrate, can then be transported to deeper parts of the aquifer.

Boxplots of nitrate concentrations in groundwater for the China and U.S. study areas are shown in Fig. 7. In the Tangshan plain region, the upper aquifer wells have the greatest range in nitrate concentrations and the highest concentration of all of the study areas, but not the highest median concentration. In fact, the median concentration in the upper part of the Tangshan plain region is less than that of the mountain aquifer and the lower part of the plain, even though over 25% of the wells sampled in the upper plain, 10% in the mountain region (mountain aquifer), and 10% in the lower aquifer (lower plain region) exceeded the Chinese standard (20 mg/l as nitrogen). Only the wells of the coastal region of the Tangshan study area (coastal aquifer) have low nitrate concentrations. All groundwater samples in this region were devoid of oxygen and, therefore, nitrate transported to the aquifer is subject to potential denitrification. Locally high concentrations of nitrate in some regions of the Tangshan study area may be attributable to wells screened just below land surface, which allows soil-applied nitrate to enter the aquifer through well bores. In the U.S. study area, nitrate concentrations in groundwater generally are lower relative to the plain region of the Tangshan study area. However, 10% of the groundwater samples of the lower aquifer in the southern San Joaquin Valley exceeded the Chinese standard; nearly 25% of the wells of the Delmarva Peninsula, and those of the upper aquifer of the western San Joaquin Valley, are close to the U.S. standard for nitrate (10 mg/l of nitrate as nitrogen) (Fig. 7); and about 18% of the samples from the eastern San Joaquin Valley exceed the U.S. standard for nitrate. Relatively few groundwater samples collected for the U.S. studies (2.6%) exceed the Chinese drinking water standard for nitrate.

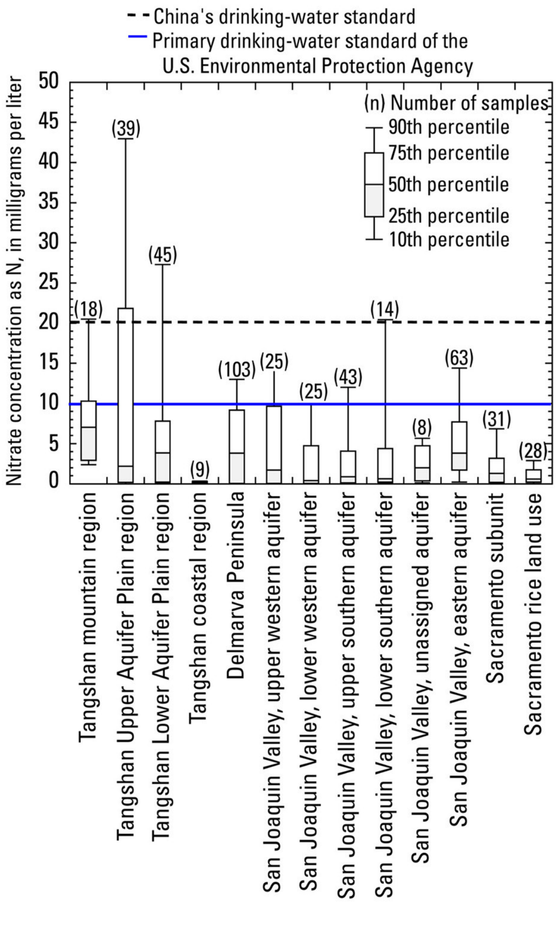

**FIGURE 7.** Boxplots of nitrate concentrations in groundwater for various zones within study areas or for an entire study area.

A comparison of nitrogen use for counties in the four study areas is shown in table 1. Data for the U.S. study areas were obtained from sales of agricultural fertilizers[14]. The Hai He River Water Conservancy Commission obtained data for the Tangshan study area through a similar process (Lin Chao, Hai He River Water Conservancy Commission, unpublished data, 1998). The nitrogen applications for some counties in the Central Valley of California are higher than those for the Delmarva Peninsula but, overall, nitrogen use per unit area in the U.S. study areas is similar (Table 1). In the counties of the Tangshan study area, nitrogen use is considerably higher, by as much as a factor of 10, than that of the U.S. study areas. Therefore, higher use of nitrogen fertilizer in the Tangshan study area is one possible explanation for the higher concentrations of nitrogen, as well as other agricultural contaminants, found in the groundwater.

Large amounts of animal manure and, in some cases, sewage, are applied to agricultural land in the Tangshan study area. As part of this study, six nitrate samples were analyzed for nitrogen and oxygen isotopes. Samples from two of the wells had a clear signature of nitrogen from fertilizer; $\delta^{15}N$ values were close to 5 per mill, typical of inorganic nitrogen fertilizer. Two samples suggested a mixed source of nitrate; $\delta^{15}N$ values were close to 10 per mill. Two samples suggested a manure or sewage source; the nitrate had a relatively high enrichment of $\delta^{15}N$, typical of manure or sewage.

Nitrate concentrations vs. well depths for the Tangshan study area are shown in Fig. 8. Nitrate concentrations to a depth of 130 to 140 m are above those expected for natural groundwater, which is consistent with the dating results. Below that depth (older water), nitrate tends to be low or below detection limits. Higher concentrations are a result of a combination of land-use practices, hydrology, biogeochemical processes, and possibly well-construction techniques. No linear relation of nitrate and well depth is apparent.

## CONCLUSIONS

A regional assessment of groundwater quality in the Tangshan region of the People's Republic of China showed that nitrate, dissolved solids, and iron are affecting the recently recharged groundwater. When compared with the groundwater in similar agricultural regions of the U.S., median nitrate concentrations tended to be similar, but a much greater range in concentrations was observed in the Tangshan study area. In the People's Republic of China, the drinking water standard for nitrate is set at 20 mg/

**TABLE 1**
**Comparison of Inorganic Nitrogen Fertilizer Use in Counties of Study Areas**

| Tangshan Study Area Counties | N Use, Mg/km²* (1996) | Delmarva Counties | N Use, Mg/km² (1991) | Sacramento Valley Counties | N Use, Mg/km² Mg/km² (1987) | San Joaquin Valley Counties | N Use, Mg/km² (1987) |
|---|---|---|---|---|---|---|---|
| Laoting | 59.1 | Kent | 5.3 | Butte | 2.4 | Fresno | 4.0 |
| Zunhua | 48.4 | New Castle | 3.0 | Colusa | 4.1 | Kern | 2.3 |
| Fengnan | 50.4 | Sussex | 3.1 | Glenn | 3.1 | Kings | 6.3 |
| Fengrun | 44.1 | Caroline | 4.9 | Placer | 0.2 | Madera | 2.4 |
| TangHai | 56.5 | Cecil | 2.7 | Sacramento | 2.5 | Merced | 4.8 |
| Luannan | 49.3 | Dorchester | 2.4 | Solano | 2.6 | San Joaquin | 7.4 |
| Yutian | 33.1 | Kent | 5.8 | Sutter | 7.9 | Stanislaus | 4.6 |
| Qianxi | 40.5 | Queen Anne’s | 5.2 | Tehama | 0.4 | Tulare | 2.9 |
| Tangshan | 36.0 | Somerset | 2.0 | Yolo | 4.8 | | |
| Luanxian | 28.5 | Talbot | 4.9 | Yuba | 2.2 | | |
| Qian an | 23.7 | Wicomico | 2.2 | | | | |
| | | Worcester | 2.0 | | | | |
| | | Accomack | 1.7 | | | | |
| | | Northampton | 2.9 | | | | |

* Mg/km² = megagram per square kilometer.

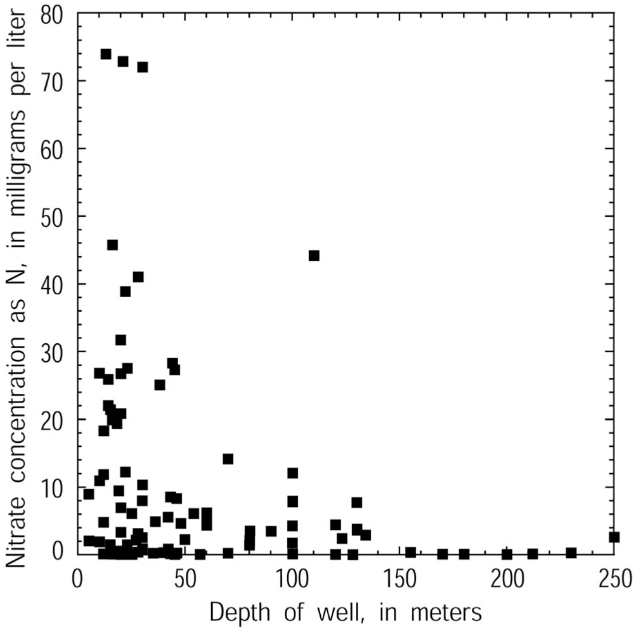

**FIGURE 8.** Nitrate concentration versus well depth for wells of the Tangshan study area.

l as nitrogen, whereas in the U.S. the standard is set at 10 mg/l as nitrogen. While many wells in the U.S. study areas exceeded the standard, only a few U.S. wells exceeded the Chinese standard. The high values of nitrate in Tangshan groundwater were attributed to larger nitrate applications, the application of animal wastes and sewage to agricultural land and land near groundwater wells, regional hydrology such as soil texture and oxidation-reduction processes, and well-construction techniques. In the Tangshan study area, groundwater ages, especially using the $^3H/^3He$ method, indicate that recently recharged water was detected to depths of as much as 150 m. This implies that agricultural contaminants could reach those depths and that in some locations deeper wells may be necessary to provide suitable drinking water. The western part of the San Joaquin Valley was shown to have similar water-quality problems, especially with respect to dissolved solids, which are elevated mainly because of the evaporation of irrigation water in a semiarid environment. In the Tangshan study area, the climate is less arid than the San Joaquin Valley, and the higher dissolved-solids content may be attributed to the longer period that the land has been in agricultural production, and to the greater amounts of land disposal of manure and animal wastes.

| | | |
|---|---|---|
| Received: | July | 11, 2001 |
| Revised: | October | 10, 2001 |
| Accepted: | October | 10, 2001 |
| Published: | November | 10, 2001 |

## BIOSKETCH

**Joseph Domagalski** is a Supervisory Hydrologist for the United States Geological Survey (Sacramento, California). He is Project Chief for Sacramento River Basin National Water Quality Assessment Program and is associated with various California projects dealing with pesticides, mercury, and organic carbon and with the Hai He River Water Conservancy Commission and Ministry of Water Resources of the People's Republic of China on a study of nutrient enrichment of reservoirs. Dr. Domagalski received his A.B. and M.S. degrees in Chemistry and Environmental Chemistry, respectively, and his Ph.D. in Geochemistry. His areas of expertise are transport and fate of organic and inorganic chemicals in surface and ground water, and use and management of pesticides in California. Research interests include transport and fate of organophosphate insecticides in surface water; transport and fate of mercury from mining areas to large rivers, and effects of management practices on mercury and other metals in surface water; nutrient enrichment of rivers and reservoirs.
